# Impact of non-agricultural experience on new farmers’ participation in e-commerce in China: A mediation analysis based on business conditions

**DOI:** 10.1371/journal.pone.0335216

**Published:** 2025-11-05

**Authors:** Liancui Wu, Haolong Yu

**Affiliations:** 1 College of Economics and Management, Zhejiang Agriculture and Forestry University, Hangzhou, China; 2 Zhejiang Institute of Rural Revitalization, Hangzhou, China; 3 Guangwen Sub-district Office, Weifang, China; University of Perugia: Universita degli Studi di Perugia, ITALY

## Abstract

In recent years, new farmers returning from non-agricultural sectors have become an important force in promoting the development of China's agricultural e-commerce. By studying the impact mechanism of non-farming work experience on their participation in e-commerce, the aim is to guide these returning new farmers to drive the development of agricultural e-commerce, improve industrial efficiency, and achieve agricultural modernization. Taking 572 new farmers returning to their hometowns in China as the target, considering the binary nature of the e-commerce participation decision (participate or not) and the continuous yet potentially censored nature of the participation degree data, the binary logistic model and Tobit model were used to analyse the influence of non-farming experience and the behaviour and degree of participation in agricultural e-commerce, and to further discuss whether there is a mediating effect of the business situation in it. The results show that: non-farming experience positively and significantly affects new farmers' e-commerce participation decision and degree, and the probability of e-commerce participation of new farmers with non-farming experience is increased by 13.5%, and the degree of their e-commerce participation is increased by 5.5%; the business situation plays a partially mediating effect; and the e-commerce participation behaviours show gender and regional heterogeneity. Based on this, suggestions are made in terms of returning home policy attraction, brand building of agricultural products, exploring foreign markets, and developing according to local conditions, so as to encourage new farmers to participate in the project of “Digital Commerce for Rural Development” and to promote rural revitalisation.

## 1. Introduction

In recent years, back home to run business and new farmers has brought new vigour and vitality to agriculture as a basic industry in China, and the study of new farmers involved in e-commerce of agricultural products is of great practical significance and reference value for developing countries in general, and especially for those countries similar to China, which are in transition from traditional to modern agriculture. The participation of new agricultural e-commerce is an important way to adapt to and develop modern agriculture in the upgrading of farmers. How to guide farmers to participate in agricultural e-commerce is also an important issue to be studied urgently. In the contemporary era, the rapid progression of modern information technology and the accelerated momentum of agricultural modernization have ushered e-commerce into the “three rural” fields thereby engendering a novel business paradigm in the information age. In 2024, the No. 1 Central Document put forth for the first time the “implementation of high-quality rural e-commerce development projects” along with the initiative to promote online sales of rural specialty products. Agricultural products e-commerce has emerged as a linchpin in fostering high-quality agricultural development and facilitating rural revitalization. It serves to alleviate agricultural production and market risks [1], augment farmers' income and entrepreneurial gains [2,3], rectify the paucity of information interaction inherent in the traditional business model [4], guarantee seamless linkages across the production, supply, and marketing chains of agricultural products, and expedite the integration of small-scale farmers into the realm of modern agriculture. According to the 2023 China Agricultural Products E-commerce Development Report, agricultural e-commerce has entered a medium- to high-speed development stage, and agricultural digital commerce has opened up new prospects for rural revitalization, driven the exploration and embrace of new agricultural industries, and catalyzed the high-quality and modern development of agriculture.

Nonetheless, the development of agricultural e-commerce is deeply affected by the low cultural level of farmers, and it is difficult for farmers to master e-commerce operation technology and management ability [5]. As the largest type of farmers in our base, traditional small farmers are deeply bound by smallholder consciousness, characterized by closedness and conservatism, which makes it difficult to integrate with e-commerce. They still use traditional ways to sell agricultural products [6]. The new farmers are different, the new farmers have received systematic knowledge education and technical training, they are open to innovation and have modern skills such as ecological concepts and Internet operations, in the era of rapid development of agricultural e-commerce can fully drive small farmers to play the role of “leading sheep” [7], leading the development of agriculture in the direction of informationization [8]. Therefore, it is of practical significance to study and analyze the participation and leadership of new farmers in agricultural e-commerce in rural areas in order to promote the project of “Digital Commerce for Agricultural Development” and realize agricultural modernization.

Previous studies have mostly focused on traditional agricultural business subjects such as ordinary farmers and agricultural enterprises [9], and few scholars have conducted research from the perspective of new farmers, who are emerging agricultural operators. Therefore, if we can focus on the new farmers to lead the further transformation and upgrading of e-commerce agriculture as a new group, and explore the path mechanism of e-commerce participation behavior and the influencing factors at a deeper level, it will not only provide theoretical basis for the realization of the in-depth integration of agriculture and e-commerce development and give full play to the leading role of the new farmers, but also help the government to formulate a more effective and accurate policy in line with the actual situation of each region, and provide a good environment for the sustainable and healthy development of rural e-commerce. It will also help the government to formulate more effective and accurate policies that are in line with the actual situation of each region, provide a favorable environment for the sustainable and healthy development of rural e-commerce, and escort the project of “Digital Commerce for Rural Development”.

## 2. Review and hypotheses

### 2.1. Literature review

Agricultural e-commerce is a business model based on Internet technology in the application of agricultural rural development, and agricultural operations to take advantage of e-commerce need to be supported by certain Internet skills, knowledge, and learning ability as a supporting element. Unlike the traditional operation mode, e-commerce is more interactive and has higher requirements for operators [10]. At present, the doctrine of new farmers “Internet+” believes that the core characteristics of the new farmers group is to have Internet thinking and be familiar with e-commerce skills [11]. It can be seen that the new farmers are able to fully utilize Internet technology to achieve the modernization of agricultural e-commerce development of the new force.

At present, in terms of the influencing factors of farmers' e-commerce adoption behavior, scholars have different priorities. First of all, individual characteristics such as educational background, age, farming experience and education [12] and their human capital [13] are important aspects affecting the e-commerce behavior of new agricultural business entities. Second, entrepreneurial training experience and self-employment experience also promote e-commerce adoption behavior [14,15]. However, there are differences in the factors influencing e-commerce participation behavior of different business entities [16]. Small farmers' willingness to adopt e-commerce is affected by their own situation, the construction of e-commerce platforms, the financing system, the improvement of the policy system [17], as well as neighborhood communication and interaction and demonstration of the role of the driving force [18]; and the willingness of new agricultural business subjects to adopt e-commerce models is affected by cognitive factors, subject factors, and operational factors [19]. In addition, industry pressure, livelihood pressure and technical expectations [20] and subjective norms [21] and other factors also have an impact on the e-commerce adoption behavior of new agricultural management subjects.

In the field of agricultural product e-commerce, scholars at home and abroad have conducted extensive research, providing multi-dimensional insights for the development of this field. However, there are certain differences in research emphases.

Chinese scholars have achieved fruitful results in the research of agricultural product e-commerce. Many studies focus on the impact of agricultural product e-commerce on various aspects of farmers. Li et al. (2024) [22] used a sample of 1229 rural households in Shandong and Shaanxi provinces in 2022 and found that e-commerce participation has a significant positive impact on the development resilience of rural households, which can enhance economic, social, and cultural resilience. Lin et al. (2024) [23], based on the 2020 China Family Panel Studies (CFPS) data, confirmed that e-commerce participation significantly promotes farmers' entrepreneurial behavior. At the same time, some studies focus on the influencing factors of farmers' participation in agricultural product e-commerce. Wei and Ruan (2022) [24], based on 554 questionnaire data from Mei County, Shaanxi Province, found that government policies and farmers' cognition affect farmers' willingness and behavior to participate in e-commerce interest-linkage mechanisms. In addition, the relationship between agricultural product e-commerce and sustainable agricultural development is also a research hotspot. Zhao et al. (2022) [25], taking the Three Gorges Reservoir area as an example, showed that e-commerce participation can promote the adoption of green agricultural technologies by reservoir resettlers. Cheng et al. (2024) [26], through the analysis of panel data of six provinces in China from 2013 to 2022, found that e-commerce participation helps to improve the environmental sustainability of transferred farmland. In terms of supply chain management, Chinese scholars have also made many explorations. Song and He (2019) [27] considered the three-layer supply chain of fresh agricultural products in the e-commerce environment, designed contract coordination mechanisms to improve supply chain performance, and found that the freshness-keeping cost-sharing and revenue-sharing contracts can achieve supply chain coordination. Liu and Yu (2023) [28] constructed a blockchain e-commerce cold-chain traceability model based on the stochastic Petri net theory to improve the reliability and effectiveness of e-commerce cold-chain traceability and promote the informatization and intelligent development of the agricultural product e-commerce supply chain. In the research on consumers' behavior in agricultural product e-commerce, Li et al. (2020) [29] took Beijing consumers as the research object, explored the key factors affecting consumers' willingness, behavior, and the consistency between willingness and behavior in purchasing agricultural products through e-commerce, and found that factors such as perceived usefulness and perceived logistics service quality have significant impacts, providing a basis for agricultural product e-commerce enterprises to optimize their services. With the development of technology, Chinese research also pays attention to the application of emerging technologies in agricultural product e-commerce.

Research on agricultural e-commerce varies by country, with some studies focusing on the development status and challenges in different regions. Oreku et al. (2013) [30] found that while Tanzania has the capability to engage in e-commerce, it needs to enhance its national image and build trust. Others focus on market mechanisms related to agricultural e-commerce: Sogn-Grundvag et al. (2024) [31] explored the relationship between product quality uncertainty, repeat purchases, and prices through auction data from Norway's pelagic fishery, while Gallenstein et al. (2021) [32] conducted experiments in rural Tanzania to study the impact of index insurance loans on farmers' credit demand and investment decisions. Considerable exploration has also been done on operational models of agricultural e-commerce platforms. For example, Quaranta and Salvia (2017) [33] launched a participatory rural development project in Campania, southern Italy, establishing a virtual agricultural e-commerce market platform to promote direct sales of local agricultural products and strengthen linkages across agricultural industry chains. In technology applications, foreign studies have deeply investigated the use of big data and artificial intelligence in market forecasting and precision marketing for agricultural e-commerce, providing technical support for market expansion and operational efficiency improvements. Supply chain optimization is central to enhancing agricultural e-commerce efficiency. In 2024, IEEE proposed an improved ant colony algorithm (IACA) [34] that, combined with blockchain technology, enables real-time temperature monitoring and energy consumption management in cold-chain logistics, reducing logistics costs by 15%. Market Data Forecast (2024) [35] predicts that AI-driven supply chain optimization will increase inventory turnover by 28%, shorten delivery times by 30%, and identify drone monitoring and smart warehousing as key growth areas.

Digital technology is becoming the core engine driving global agricultural modernization and rural economic transformation, particularly in developing countries, where its impact on agricultural production efficiency, e-commerce penetration, and sustainable development has become increasingly significant. Existing research, focusing on technology adoption, governance mechanisms, social capital, and regional practices, reveals a complex landscape of opportunities and challenges. Digital technology plays a pivotal role in global agricultural modernization and rural economic transformation, with notable effects in developing countries. A study in Pakistan shows that internet technology has increased the technical efficiency score of wheat farmers by 12.7%, with more significant improvements observed among less efficient farmers (Ahmad et al., 2024) [36]. In China, rural e-commerce faces governance challenges arising from information asymmetry, which require optimization through service evaluation systems and social capital building (Cai et al., 2019) [37]. Meanwhile, farmers' adoption of e-commerce is influenced by multiple factors, including gender, policy perception, and infrastructure (He et al., 2024) [38]. The optimization of the digital environment has a positive effect on farmers' entrepreneurial behavior, but there is an inverted U-shaped relationship between digital literacy and digital rural development, necessitating a balance between hardware and soft environment construction (Chen et al., 2024) [39].The association between social capital types and sustainable development exhibits regional disparities. For example, the social capital composition of rural poor groups in Malawi differs from traditional models (Craig et al., 2022) [40], while the development of rural e-commerce in BRICS countries is unbalanced, calling for strengthened transnational policy coordination (Haji, 2021) [41]. In technological innovation, blockchain enhances the transparency of agricultural product traceability systems but remains costly (Demestichas et al., 2020) [42]. In Nigeria, digital platforms have improved supply chain efficiency by 25%, yet this is constrained by limited internet coverage (Sanusi et al., 2025) [43]. Online procurement decisions of agricultural enterprises in Germany indicate that price discounts, certification labels, and delivery timeliness are key influencing factors (Danne et al., 2021) [44], while China has achieved e-commerce innovation in poverty-stricken areas through resource orchestration theory (Cui et al., 2017) [45].

Overall, domestic research closely integrates with the actual development of domestic agriculture, focusing on the impacts of e-commerce on farmers' individual development, agricultural production, and rural development, as well as relevant policy recommendations. The research covers aspects such as the enhancement of development resilience, promotion of entrepreneurial behavior, adoption of green agricultural technologies, and improvement of environmental sustainability, while also conducting in-depth explorations in supply chain management, consumer behavior, and applications of emerging technologies. Research in other countries, by contrast, emphasizes an international perspective, focusing on the challenges in e-commerce development across countries with different development levels, such as trust-building and infrastructure constraints and the micro-mechanisms of e-commerce in agricultural product markets, including quality uncertainty and repeat purchase behavior. It also examines the linkages between these mechanisms and agricultural elements such as technological innovation (e.g., big data and artificial intelligence in market forecasting and precision marketing) and supply chain optimization (e.g., real-time cold-chain logistics monitoring and blockchain traceability systems).

While existing literature widely discusses the potential of new farmers in driving rural e-commerce development through technological applications and “Internet+” thinking, it lacks an in-depth exploration of specific policy mechanisms to effectively mobilize their enthusiasm, such as training systems, financing support, and incentive measures. At the rural development level, although existing literature recognizes the importance of rural economic development and the implementation of the rural revitalization strategy, it has not fully analyzed the specific pathways through which new farmers promote rural economic development by expanding external markets (e.g., through cross-regional e-commerce platform integration) and advancing agricultural product brand building (e.g., via standardization certification and brand marketing). Encouraging new farmers to expand market boundaries and shape brand value through e-commerce platforms can create new growth drivers for rural economies by increasing product premium and enhancing market competitiveness, thereby empowering income growth for farmers and deepening the implementation of the rural revitalization strategy.

This study fills the above gaps by analyzing the practical paths of new farmers' market expansion behaviors and agricultural product branding, providing a more complete theoretical framework for understanding the interactive logic of “new farmers-industrial upgrading-rural revitalization.” Future research should strengthen the integration of Chinese and foreign research paradigms and expand the research boundaries of agricultural e-commerce in emerging fields such as digital rural construction and climate change adaptation, offering theoretically profound and practically oriented solutions for global agricultural digital transformation.

### 2.2. Research Hypotheses

#### 2.2.1. Non-farm experience.

Compared with traditional farmers, new farmers have rich experience, young and highly educated characteristics [46], for new things, new ideas acceptance willingness and ability is stronger. Labor experience is conducive to enriching the social capital of farmers (Xu, C. et al., 2017) [47]. Outside workers have a sharper sense of information and a stronger ability to accept new things [48], which determines that new farmers with labor experience have a higher degree of acceptance of agricultural e-commerce. The process of migrant labor in turn brings about the re-accumulation of human capital, including the updating of knowledge structure, the acquisition of new technologies, and the change of ideological concepts [49]; the experience of doing business can bring about the improvement of social capital (Wang, X. and Gao, Y., 2017) [50]. In addition, business experience makes economic capital improve (Feng, H., 2016) [51]. Work and business experience can also broaden the horizons, and new farmers with business experience are more likely to find new opportunities for agricultural development. Improved economic capital provides financial support for new farmers' agricultural e-commerce entrepreneurship.

Comprehensively, it seems that the experience of working and doing business is conducive to the accumulation of social capital, human capital and economic capital of farmers, and new farmers with non-agricultural experience of working and doing business have a stronger willingness to participate in agricultural e-commerce and the ability to participate in agricultural e-commerce, and they are more inclined to choose agricultural e-commerce entrepreneurship.

Rich previous non-farming experience makes new farmers more willing to accept agricultural e-commerce. Non-farming experience allows new farmers to accumulate a certain amount of human, financial, and social capital, and all of these accumulations can significantly contribute to e-commerce adoption behavior [52]. First, the experience of working in the field makes new farmers more willing to accept agricultural e-commerce. With higher professional skills and innovative thinking, new farmers are more willing to accept and adopt agricultural e-commerce channels for sales than traditional farmers. Secondly, new farmers are more capable of applying e-commerce technology to agriculture. Previously, they had different experiences in the city, which enabled them to complete the accumulation of capital and other primitive capital for the field of agricultural e-commerce [53]. In addition, their non-farming experience gives them a wider range of social networks and resource channels, more fluid access to information, and easier access to market information so that they can find the market and consumer demand points. Therefore, the following hypothesis H1 is proposed:

**H1:** *Non-farming experience has a positive impact on new farmers' e-commerce participation*

**H1a:** *Non-farming experience has a positive impact on new farmers' e-commerce engagement behavior*

**H1b:** *Non-farming experience has a positive impact on the level of e-commerce participation of new farmers*

#### 2.2.2. Business status.

In addition, there is also a correlation between new farmers' business characteristics and e-commerce adoption behavior. In previous studies, different subjects with different business status showed different attitudes towards e-commerce adoption behavior [54]. In previous studies, the different business status of different subjects showed different attitudes towards e-commerce adoption behavior. The degree of market exposure and branding of agricultural products can reflect the business status of new farmers. Generally speaking, the better the business efficiency of new farmers, the more they want to broaden their sales channels. Branded agricultural products of new farmers oriented to foreign markets are more in need of broadening sales channels, and therefore more willing to adopt e-commerce participation. Accordingly, this paper proposes H2:

**H2:** *Business status affect e-commerce adoption behavior of new farmers*

**H2a:** *Market type has an impact on the e-commerce participation behavior of new farmers, and exploring foreign markets promotes e-commerce participation behavior*

**H2b:** *Brand building has an impact on the level of e-commerce engagement of new farmers, and brand building promotes e-commerce engagement behavior*

#### 2.2.3. Intermediation effects.

In terms of the relationship between non-farming experience, business situation and e-commerce participation, non-farming experience implies more open-mindedness and rich capital accumulation, compared with new farmers without non-farming experience, new farmers with non-farming experience have the advantage of human connections and wealth accumulation, can apply their modern business thinking to agricultural production and management, are willing to improve the quality of agricultural products to realize brand management and develop. The new farmers are willing to improve the quality of agricultural products to realize brand management and explore the market of agricultural products from outside to enhance the operating efficiency, and will further adopt the behavior of e-commerce participation. Accordingly, this paper proposes the following research hypothesis H3.

**H3:** *Non-farming experience affects new farmers' e-commerce adoption behavior through business characteristics*

**H3a:** *Non-farm experience promotes e-commerce adoption behaviors among new farmers by exploring foreign markets*

**H3b:** *Non-farm experience promotes e-commerce adoption behaviors among new farmers by facilitating brand building*

**Fig 1:**
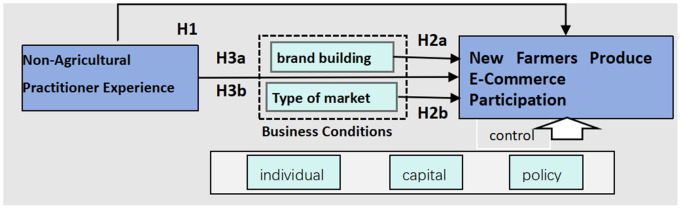
Initial conceptual model.

Based on this, this study focuses on the influence of new farmers'’ non-farming experience and business situation on the adoption behavior of agricultural e-commerce. In addition, referring to the focus of previous scholars and combining with the above analysis, control variables are selected from the individual characteristics of new farmers, capital situation, and policy environment to be included in the study, and the specific influence mechanism is shown in the Fig 1 below:

## 3. Materials and methods

### 3.1. Data sources

The research data in this paper comes from the National Social Science Foundation Program-Research on the Benefit Linkage Mechanism between New Farmers and Small Farmers Based on the Benefit Sharing Perspective. In order to ensure the representativeness of the sample and the generalizability of the results obtained from the data analysis, the research team used random stratified sampling of new farmers in six provinces in China from 20/07/2021-20/09/2021. The use of the stratified random sampling method allows for stratification and sample selection based on different characteristics such as geographical location, economic development level, agricultural production type, and socio-cultural background. This comprehensively reflects the diversity of the overall population, enhancing the universality and representativeness of research results. Moreover, it can reduce sampling errors. By minimizing the within-stratum variance, it improves the accuracy of the estimation of overall parameters, providing a more accurate basis for policy-making. Additionally, it facilitates subsequent research analysis. It enables in-depth analysis of samples from different strata respectively, uncovering more valuable information. When resources are limited, the focus can be placed on key strata, sampling resources can be allocated rationally, the sample size can be reduced, and it helps to complete high-quality research under limited conditions.

In the central region, Henan Province, known as the “Breadbasket of the Central Plains,” has a well-developed agricultural sector and a large agricultural population, and has been experiencing a sustained agricultural development trend in recent years, as well as a successful development of modern agriculture. Anhui is located in the Jianghuai land, the natural conditions of agricultural production is excellent, is a typical agricultural province. Therefore, the research group selected Henan, Anhui as the central region of the research object; in the eastern region, since the reform and opening up, Jiangsu and Zhejiang region, the sustained and healthy development of the economy, in the country's economic development, leading and driving the development of the surrounding areas and even the national economy, very research value; in the western region, known as the country of the capital of Sichuan, a strong foundation for agriculture, agricultural production conditions are superior, and Gansu Province is located in the northwestern part of China, the climate type is rich and the regional terrain is very good. Climate type rich and complex terrain across the region, complex geographical conditions make its agricultural development with diversity, so the research group selected Sichuan, Gansu as the western research object.

For the data in Zhejiang Province, the research group mainly used field research, with the assistance and support of the Zhejiang Provincial Department of Agriculture, visited new farmers in various areas of the province, asked in detail, researched and recorded the data, and audited and proofread the data to ensure that the data were accurate and true; for the data outside Zhejiang Province, the research group, with the help of the provincial departments of agriculture, used the form of the network questionnaire of the WeChat link to fill out the questionnaires through cell phones or computer equipment to fill in. The researchers sent each questionnaire to the hands of the new farmers around the world and dispatched professional assistants to help the new farmers to fill in the questionnaires, for the questionnaires retrieved, the research team and the professional assistants cooperated in the review and proofreading to ensure that the questionnaire data is true and reliable. A total of 674 questionnaires were distributed in this research, and after the questionnaire information was summarized and processed, a total of 572 valid questionnaires could be applied to this study. Among the valid questionnaires, 216 were from the central part (Anhui Province and Henan Province), 182 were from the eastern part (Zhejiang Province and Jiangsu Province), and 174 were from the western part (Sichuan Province and Gansu Province), as shown in Table 1 below. In conclusion, the sampling and data collection was launched in full swing and successfully completed in 2021, laying a solid data foundation for this research.

**Table 1:** Data sources and sample distribution.

Region	Selected Provinces	Reasons for Selection	Questionnaires Distributed	Valid Questionnaires
**Central**	Henan, Anhui	Henan: Well-developed agriculture, large agricultural population, modern agriculture success. Anhui: Typical agricultural province with excellent natural conditions.	238	216
**Eastern**	Jiangsu, Zhejiang	Sustained economic development, leading role in national economy and regional driving effect.	220	182
**Western**	Sichuan, Gansu	Sichuan: Strong agricultural foundation, superior production conditions. Gansu: Diverse climate and terrain, agricultural development diversity.	216	174
**Total**	–	–	674	572

Data source: research data collation, similarly hereinafter

### 3.2. Variable descriptions

#### 3.2.1. Explanatory variable.

The core explanatory variables of this study are agricultural e-commerce participation behavior and level of participation.

This paper refers to agricultural e-commerce as distinct from agricultural e-commerce and rural e-commerce [55], which refers to e-commerce activities in the supply chain link after the production of agricultural products [56] In the questionnaire, the questionnaire asked “Are agricultural products sold through e-commerce? In the questionnaire, the questionnaire is expressed as “whether the agricultural products are sold through e-commerce?” “Percentage of e-commerce sales”. The agricultural sales platforms include both traditional e-commerce platforms such as WeChat Small Program, WeChat Circle of Friends, Taobao, Jingdong(JD), Pinduoduo(PDD), Meituan and other e-commerce platforms, as well as e-commerce platforms formed by local governments.

**Fig 2:**
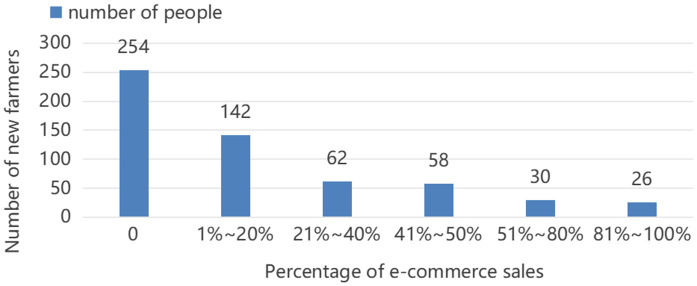
Distribution of the proportion of e-commerce sales of agricultural products by new farmers.

Fig 2 gives the participation of new farmers in e-commerce sales of agricultural products. It can be found that among a total of 572 new farmers studied, 254 new farmers did not adopt e-commerce sales behavior, accounting for about 44.4%. Among the 318 new farmers who adopted e-commerce sales of agricultural products, only 56 new farmers used e-commerce sales as their main sales channel. More than 80% of the new farmers' e-commerce sales channels accounted for less than 40% of all channels. Therefore, it can be concluded that although more than half of the new farmers have adopted e-commerce sales of agricultural products, the degree of participation in e-commerce of agricultural products by new farmers is still not high.

**Fig 3:**
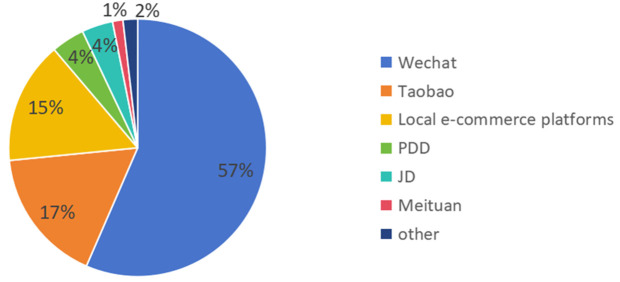
Use of e-commerce sales platforms.

In the use of e-commerce sales platforms counted in Fig 3, it can be seen that the WeChat platform is the most dominant platform for the e-commerce activities of new farmers. Compared to other e-commerce platforms, the main advantage of WeChat platform is its wide coverage of user volume. As a national social product, WeChat platform has 1.327 billion users. E-commerce small program backed by WeChat social traffic dividend, can be in the WeChat ecosystem through the circle of friends, community sharing, complete social fission. Moreover, the use of WeChat e-commerce platform can be used directly through the WeChat portal without the need to additionally download and install other APPs, and there is no additional platform rent, so the cost of using the platform is lower. Therefore, WeChat has become the most popular e-commerce platform for new farmers. In addition, the types of e-commerce platforms used by new farmers are also diversified, not only traditional e-commerce platforms such as Taobao, Jingdong and Pinduoduo, but also local e-commerce platforms supported by local governments such as Jitterbug, micro store and so on.

#### 3.2.2. Core explanatory variables.

The core explanatory variable of this study is non-farming experience. In this study, the new farmers with the experience of “purely working in agriculture” = 0; the new farmers with the experience of “having worked in state-owned enterprises, enterprises and public institutions, private enterprises, and freelancing” = 1. The sample statistics show that 74.6% of the new farmers interviewed had non-farming experience before adopting the e-commerce sales of agricultural products. E-commerce sales before adopting non-farming experience.

New farmers come from all walks of life and have a wealth of previous experience in the industry [53]. Among the sample of new farmers surveyed, more than 60% of them have never been involved in agricultural production before, and only 37.2% of them have been engaged in agricultural activities before. More than 40% of the new farmers were once freelancers; and only 8.7% of the new farmers returned to their hometowns to start their own businesses after graduating from university.

In addition, as reflected by the data in Table 2, new farmers maintain more of an adoption attitude towards e-commerce sales behavior. In addition to the proportion of new farmers with farming experience adopting e-commerce is less than 50%, the adoption of e-commerce sales behavior by new farmers with other experience is generally around 60%. Therefore, it can be seen that compared with purely farming new farmers, new farmers with rich experience in the field are more inclined to adopt the e-commerce behavior of agricultural products.

**Table 2:** E-commerce adoption behavior of new farmers with different employment experiences.

Previous experience	Adoption of e-commerce sales					
	Yes		No		grand total	percentage (all new farmers)
	number of people	percentage	number of people	percentage		
farming	103	48.4%	110	51.6%	213	37.2%
State-run, enterprises and institutions	44	55.0%	36	45.0%	80	14.0%
private business	112	67.1%	55	32.9%	167	29.2%
profession	133	55.2%	108	44.8%	241	42.1%
Graduated students	34	68.0%	16	32.0%	50	8.7%

#### 3.2.3. control variable.

Drawing on the research results of existing studies, this study will select control variables from the aspects of individual characteristics [57,58], capital investment [59], and policy environment of new farmersu [60]: 1) For individual characteristics, five variables are selected, namely, gender, age, political appearance, and whether or not to participate in cooperatives and agricultural insurance cognition; 2) For capital investment, four variables are selected, namely, years of entrepreneurial experience, the proportion of agricultural income, capital investment, and labor input; 3) For the aspect of policy environment, whether or not to obtain support is selected, difficulty of policy access variables; 4) For region, the central region is taken as the benchmark, and dummy variables for the eastern and western regions are set. The specific definitions of the explained variables, explanatory variables and control variables involved in this study are shown in Table 3.

**Table 3:** Meaning of variables and their values.

Variable type	variable name	Variable Definition and Assignment	average value	(statistics) standard deviation
implicit variable	E-commerce participation behavior	Adoption of e-commerce sales of agricultural products?1 = Yes; 0 = No	0.556	0.497
	Level of e-commerce participation	Percentage of agricultural e-commerce sales channels among all channels?	0.200	0.266
independent variable	Non-Agricultural Practitioner Experience	Prior to becoming a new farmer, was there ever any non-farming experience? 1 = yes; 0 = no	0.747	0.435
individual characteristic control variable	distinguishing between the sexes	1 = male; 0 = female	0.740	0.439
	(a person's) age	Unit: weeks of age	42.781	10.179
	political profile	Are you a member of the party? 1 = Yes; 0 = No	0.322	0.468
	Agricultural Insurance Awareness	Perceived importance of agricultural insurance?1 = greater; 0 = considerable or lesser	2.030	1.202
	Participation in cooperatives	Participation in agricultural cooperatives? 1 = Participation; 0 = Non-participation	0.591	0.492
Capital input control variables	years of experience	How many years is a farm operation?	9.552	7.956
	Share of income from agriculture	Share of agricultural income in total household income?	0.819	0.271
	capital investment	What was the total amount of money you invested in agriculture last year? Take the ln value	4.411	1.226
	labor input	What was the total number of actual agricultural labor inputs last year? Take the value of ln	2.453	1.377
policy environment control variable	Availability of support	Does e-commerce sales receive policy support?1 = Yes; 0 = No	0.804	0.397
	Difficulty in accessing policies	Ease of obtaining entrepreneurial support from government departments, very easy = 1; easier = 2; average = 3; more difficult = 4; very difficult = 5	2.126	0.986
intermediary variable	brand status	Have you created a brand for your agricultural products? 1 = Yes; 0 = No	0.491	0.500
	market segment	The main product markets for the agricultural products you produce? 1 = field market; 0 = local market	0.379	0.486
virtual variable	eastern part	1 = in the East; 0 = not in the East	0.318	0.466
	western region	1 = in the West; 0 = not in the West	0.304	0.460

### 3.3. Model construction

#### 3.3.1. Binary logistic model.

Logistic model is widely used by scholars to conduct research related to the factors influencing the behavior of agricultural subjects. Therefore, this study uses a binary Logistic model to analyze the influencing factors of the behavior of new farmers' e-commerce participants. The modeling is established as:


$$P=F_{\left(Y\right)}=\frac{\text{1}}{1+e^{-Y}}$$ (1)

In Eq. (1), P represents the probability that new farmers choose to participate in agricultural e-commerce; Y represents e-commerce participation behavior; Y=1 represents that new farmers participate in agricultural e-commerce; Y=0 represents that new farmers do not participate in agricultural e-commerce. The linear combination of Y core independent variable X and each control variable $C_{i}(i=1,2,...,n)$ in Eq. (1), $C_{i}$ represents the influencing factors of new farmers' e-commerce participation behavior, i.e.,:


$$Y=\beta_{0}+\beta_{X}X+\beta_{1}C_{1}+\beta_{2}C_{2}\cdot \cdot \cdot +\beta_{n}C_{n}$$ (2)

In Eq. (2), $\beta_{\text{0}}$ is the constant term; $\beta_{X}$ is the regression coefficient of the core independent variable X, and $\beta_{i}(i=1,2,...,n)$ is the regression coefficient of thei th control variable. Equation (1) (2) is processed to obtain the binary logistic model as:


$$logit\left[\frac{p}{1-p}\right]=\beta_{\text{0}}+\beta_{X}X+\beta_{\text{1}}C_{1}=\beta_{2}C_{2}+\cdot \cdot \cdot +\beta_{n}C_{n}+\varepsilon$$ (3)

In equation (3), ε is the random error term. The paper utilized Stata15.1 to regress the survey data on a binary logistic model.

#### 3.3.2. Tabit model.

When studying the participation of new farmers in agricultural product e-commerce, the data exhibits obvious censoring characteristics. In the sample, 254 new farmers did not participate in e-commerce, accounting for approximately 44% of the total sample. Their degree of e-commerce participation is valued at 0, which belongs to censored data with a left-hand censoring point of 0.

The ordinary least squares (OLS) method is suitable for data where the dependent variable is continuously distributed. It assumes that the error term meets conditions such as normal distribution and homoscedasticity. However, for the censored data in this study, OLS treats the censored observations with a value of 0 the same as non-censored observations, ignoring the underlying mechanisms behind the 0 values. This leads to biased estimation results, fails to accurately reflect the relationships between variables, and reduces the accuracy and reliability of the model's predictions.

The Tobit model is specifically designed to handle data with a censored dependent variable [61]. It divides the generation process of the dependent variable into two parts: a latent continuous variable and an observed censoring process. This model can comprehensively consider the situation where the degree of new farmers' e-commerce participation is 0. Through maximum likelihood estimation for analysis, it can obtain unbiased and efficient parameter estimates, accurately analyze the influence of various factors on the degree of e-commerce participation, and provide a reliable basis for decision-making. Therefore, based on the censoring characteristics of the data and the limitations of the OLS method, it is more appropriate to use the Tobit model for regression [62]. The econometric model is as follows:


$$TobitE_{i}=\beta_{0}+\beta_{k}X_{k}+\beta_{j}X_{j}+\varepsilon$$ (4)

In Eq. (4): $E_{i}$ is the level of e-commerce participation of new farmers; $X_{k}$ is the non-farm experience variable; $X_{j}$ is the control variable; $\beta_{k}$ and $\beta_{j}$ are the estimated coefficients of the core independent variable and the control variable, respectively; $\beta_{\text{0}}$ is the model intercept term; and ε is the random error term.

#### 3.3.3. Mediated effects modeling.

In order to verify the mediating path of business status in the non-farming experience affecting the e-commerce participation behavior of new farmers, this paper refers to the causal effect stepwise regression method proposed by Baron and Kenny to test the mediating variables [63], which establishes the following regression equation for testing.


$$Y_{i}=\alpha_{1}X_{1}+\beta_{2}C_{i}+\delta_{i}$$ (5)

$$M_{i}=\alpha_{2}X_{i}+\beta_{2}C_{i}+\varepsilon_{i}$$ (6)

$$Y_{i}=\alpha_{3}X_{i}+\alpha_{4}M_{i}+\beta_{3}C_{i}+\varepsilon_{i}$$ (7)

In the regression equation, $Y_{i}$ is the dependent variable of new farmers' agricultural e-commerce participation behavior, $X_{i}$ is the independent variable of non-farming experience, Mi is the mediating variable of business status (market-oriented type, building agricultural brands), $C_{i}$ is the control variable, α and β are the parameters to be estimated, and $\varepsilon_{i}$ is the random disturbance term. Eq. (5) is the total effect of the independent variable $X_{i}$ on the dependent variable $Y_{i}$, Eq. (6) is the configuration effect of the independent variable $X_{i}$ on the mediator variable $M_{i}$, and Eq. (7) is the effect of the independent variable $X_{i}$ on the dependent variable$Y_{i}$ under the influence of the control mediator variable $M_{i}$. Among them, Eqs. (5), (6) and (7) are regressed using binary logistic model for regression.

## 4. Results and discussion

### 4.1. Regression analysis

#### 4.1.1. The impact of non-farming experience on the e-commerce engagement behavior of new farmers.

Given that there may be internal correlation between the explanatory variables, if the correlation is serious, it will lead to a large bias in the estimation results. Therefore, in order to avoid the problem of covariance between the explanatory variables, before the regression, the variance inflation factor VIF is used to carry out the test of multicollinearity, and the test results show that: Mean VIF = 1.32, and the VIF of each indicator is less than 1.80, which is much smaller than the critical value of the existence of multicollinearity, so it can be determined that the degree of covariance between the explanatory variables is in the range of reasonableness, and it can be carried out in the next step. Empirical analysis.

In the benchmark regression, in order to ensure the robustness of the regression results, discover the changes in the core variables after adding different control variables in a timely manner, and avoid the situation in which changes or even the opposite sign are highly likely to occur after adding different control variables, the core explanatory variables, individual characteristic-related variables, capital inputs, the policy environment, and the regional dummy variables are added in this way in the benchmark regression. In the regression results in Table 4, the Pseudo R2 values in Models 1–5 are gradually improved, with P-values all significant at the 1% statistical level, and the core explanatory variables have the same direction of influence, with the significance changing slightly overall, suggesting that the model estimation results have strong robustness. In addition, in order to better explain the regression results, the regression that incorporates all variables (model 5) is treated with marginal effects.

**Table 4:** Impact of non-farming experience on e-commerce engagement behavior of new farmers.

variable name	Model 1	Model 2	Model 3	Model 4	Model 5	marginal effect (Model 5)
Non-Agricultural Practitioner Experience	1.858*** (0.360)	1.663** (0.335)	1.757*** (0.370)	1.807*** (0.384)	1.819*** (0.389)	0.135***
distinguishing between the sexes		1.399* (0.283)	1.368 (0.281)	1.343 (0.278)	1.354 (0.281)	0.068
(a person's) age		0.972*** (0.009)	0.968*** (0.010)	0.964*** (0.010)	0.964*** (0.010)	−0.008***
political profile		1.138 (0.215)	1.173 (0.227)	1.126 (0.219)	1.144 (0.224)	0.030
Agricultural Insurance Awareness		0.935 (0.067)	0.937 (0.068)	0.960 (0.072)	0.962 (0.072)	−0.009
Participation in cooperatives		1.594** (0.286)	1.309 (0.257)	1.253 (0.249)	1.256 (0.250)	0.051
years of experience			1.010 (0.013)	1.012 (0.013)	1.012 (0.013)	0.003
Share of income from agriculture			1.997** (0.672)	1.988** (0.670)	2.054** (0.699)	0.163**
capital investment			1.251*** (0.103)	1.262*** (0.105)	1.292*** (0.116)	0.058***
labor input			0.963 (0.073)	0.955 (0.073)	0.965 (0.076)	−0.008
Availability of support				1.688** (0.397)	1.678** (0.395)	0.117**
Difficulty in accessing policies				0.854* (0.078)	0.845* (0.079)	−0.038*
constant term (math.)	0.790 (0.132)	1.876 (0.890)	0.508 (0.317)	0.538 (0.365)	0.420 (0.318)	–
Area dummy variables	uncontrolled	uncontrolled	uncontrolled	uncontrolled	Controlled	–
Pseudo R2	0.013	0.038	0.055	0.064	0.065	–
Prob > chi2	0.001	0.000	0.000	0.000	0.000	–

Note: *, **, *** indicate significant at the 10%, 5% and 1% statistical levels, respectively. Standard errors in parentheses, same below.

Non-farming experience has a significant positive effect on new farmers' e-commerce participation behavior, which is significant at the 1% statistical level. The probability of the occurrence of agricultural e-commerce participation behavior of new farmers with non-farming experience is increased by 13.5%, and the estimation results of Models 1–3 are also significant, thus confirming Hypothesis **H1a**, that is, non-farming experience directly promotes the agricultural e-commerce participation behavior of new farmers. The reason is that the new farmers' previous experience in secondary and tertiary industries has enabled them to obtain sufficient primary capital accumulation and gain more experience and skills in e-commerce operation, marketing, supply chain management, etc., so that they can accept e-commerce sales channels by recognizing the advantages and prospects of e-commerce for agricultural products.

In terms of individual characteristics, new farmers' gender, age, and whether they participate in cooperatives affect their e-commerce participation behavior. The age of new farmers significantly affects the e-commerce participation behavior of agricultural products, which is significant at the 1% level, probably because a large number of college students returning to their hometowns to start their own businesses use the e-commerce skills they have learned to apply them to the sales side of the agricultural supply and form a group of new forces to promote the development of e-commerce for agricultural products. In addition, male, new farmers involved in cooperatives are more inclined to develop e-commerce channels for agricultural sales.

In terms of capital investment, the share of agricultural income and capital investment of new farmers have a significant positive impact on participation in the e-commerce sales of agricultural products. Adequate capital investment in agriculture can improve the yield and quality of agricultural products through the introduction of modern agricultural equipment and technology, which in turn increases the supply and market value of agricultural products. Higher-quality agricultural products satisfy more consumer demand, and thus new farmers choose to adopt e-commerce marketing channels for agricultural products to broaden new marketing channels to realize business benefits. The higher proportion of agricultural income of new farmers means that new farmers' entrepreneurship is bigger and stronger, and the local market cannot meet their development needs. And agricultural products e-commerce sales, as a new sales model, has the advantages of price transparency, extensive sales channels, consumer feedback in a timely manner, etc. These advantages, as well as the demand for a broad field market, make new farmers choose e-commerce sales channels.

With regard to policy support, access to e-commerce policy support can significantly and positively influence the decision of e-commerce participation behavior and is significant at the 5% level. The difficulty of support acquisition is significantly positively promoting e-commerce participation behavior at the 10% level, and the easier it is to obtain policy support, the more inclined new farmers are to e-commerce participation. It can be seen that creating a favorable e-commerce policy environment can promote the participation of new farmers in agricultural e-commerce.

In conclusion, the empirical analysis shows that the non-agricultural experience has a significant positive impact on the e-commerce participation behavior of new farmers, and the individual characteristics, capital investment and policy aspects all influence the decision of new farmers to engage in the e-commerce of agricultural sales.

#### 4.1.2. the impact of non-farming experience on the level of e-commerce participation of new farmers.

Does the non-farming experience of new farmers affect their e-commerce participation? To address this question, a Tobit model was used to regress the data. To ensure the robustness of the regression results, the core explanatory variables, individual characteristic-related variables, capital investment, policy environment and regional dummy variables are added to the model in turn.6–10 In addition, the Tobit model coefficients cannot directly explain the probability of occurrence of the explanatory variables, so at the same time, by solving for the average marginal effect of each variable of the Tobit model, the contribution of the independent variables to the degree of e-commerce participation is examined. participation level contribution, and the results are shown in Table 5.

**Table 5:** Impact of non-farming experience on e-commerce participation of new farmers.

variable name	Model 6	Model 7	Model 8	Model 9	Model 10	marginal effect (Model 10)
Non-Agricultural Practitioner Experience	0.117*** (0.045)	0.090** (0.045)	0.095** (0.046)	0.096** (0.046)	0.099** (0.046)	0.055**
distinguishing between the sexes		0.084* (0.044)	0.075* (0.044)	0.073* (0.044)	0.075* (0.044)	0.042*
(a person's) age		-0.006*** (0.002)	-0.008*** (0.002)	-0.008*** (0.002)	-0.008*** (0.002)	-0.004***
political profile		-0.045 (0.041)	-0.051 (0.040)	-0.054 (0.041)	-0.053 (0.041)	-0.029
Agricultural Insurance Awareness		0.004 (0.016)	0.006 (0.015)	0.007 (0.016)	0.007 (0.016)	0.004
Participation in cooperatives		0.111*** (0.039)	0.054 (0.042)	0.050 (0.042)	0.053 (0.042)	0.029
years of experience			0.005* (0.003)	0.005* (0.003)	0.005* (0.003)	0.003*
Share of income from agriculture			0.119* (0.070)	0.118* (0.070)	0.123* (0.071)	0.068*
capital investment			0.051*** (0.017)	0.052*** (0.017)	0.052*** (0.019)	0.029***
labor input			0.002 (0.016)	0.001 (0.016)	0.004 (0.016)	0.002
Availability of support				0.027 (0.050)	0.026 (0.050)	0.014
Ease of access to policies				-0.022 (0.019)	-0.022 (0.019)	-0.012
constant term (math.)	0.790 (0.132)	0.127 (0.103)	-0.141 (0.132)	-0.107 (0.142)	-0.145 (0.158)	-
Area dummy variables	uncontrolled	uncontrolled	uncontrolled	uncontrolled	Controlled	-
Pseudo R2	0.009	0.035	0.060	0.062	0.063	-
Prob > chi2	0.001	0.000	0.000	0.000	0.000	-

The regression results presented above indicate that, in comparison to new farmers solely engaged in agricultural activities, those with prior non-farm experience exhibit a 5.5% higher level of participation in e-commerce. Evidently, non-farm experience exerts a significant positive influence on the extent of new farmers’ e-commerce participation, with this impact being statistically significant at the 5% level. Consequently, Hypothesis **H1b** is empirically validated. In terms of individual characteristics, gender and age of new farmers are important factors affecting the degree of e-commerce participation. Young male new farmers have a higher degree of e-commerce participation. In addition, in terms of capital investment, the proportion of capital investment and agricultural income and the number of years in business affect the degree of e-commerce participation of new farmers to a certain extent, and the degree of e-commerce participation is higher among new farmers with more capital investment, a higher proportion of agricultural income, and a higher number of years in business.

### 4.2. Endogeneity test-based *on* PSM modeling

New farmers' non-farming practice behavior is the result of self-selection, in order to avoid the problem of sample selection bias, the article uses the propensity score matching (PSM) method for endogeneity treatment. Pure farming new farmers as the treatment group (treat = 1) and new farmers with non-farm practice experience as the control group (treat = 0), then the nearest-neighbor 1:1 matching method is used for pairing and sample no-return matching for selected observable variables, and the propensity scores are estimated by the Logit model, and the results of the treatment are shown in the following table. The results are shown in Table 6, the t-value of the average treatment effect (ATT) for the treatment group is 2.36, which is greater than 1.96 and passes the test of significance at the 5% level.

**Table 6:** PSM treatment results.

variant	sample matching		process group	control group	difference (the result of subtraction)	standard error	T-value
Whether or not to engage in e-commerce practices	prematch		0.595	0.441	0.153	0.0474	3.24
	after matching	ATT	0.579	0.441	0.138	0.0583	2.36
		ATU	0.441	0.566	0.124		
		ATE			0.131		

The matching results show that the standardized deviations of the variables are all less than 10%, and the results of the t-test do not reject the original hypothesis that there is no systematic difference between the treatment group and the control group; at the same time, the matching results satisfy the common support hypothesis. Regression is conducted using the sample after PSM to explore the impact of non-farming experience on the e-commerce participation behavior of new farmers after excluding the endogeneity problem caused by sample selection bias. According to the regression analysis in Table 7, it can be seen that non-farming experience has a significant positive impact on new farmers' e-commerce participation behavior. This is consistent with the previous findings, indicating that the research conclusions still hold after considering possible endogeneity problems. In conclusion, through the endogeneity test based on the PSM modeling, we effectively addressed the potential sample selection bias issue. The results demonstrate that after treatment, non-farming experience still has a significant positive impact on new farmers' e-commerce participation behavior, which is consistent with the previous research findings.

**Table 7:** PSM sample regression results.

variant	E-commerce participation behavior	
	OR value	standard error
Non-Agricultural Practitioner Experience	1.864**	0.531
control variable	Controlled	
observed value	246	
R-squared	0.087	

### 4.3. Mediating effect test

In order to verify whether non-farming experience indirectly affects e-commerce participation behavior through the business status variable, this paper empirically tests the mediation effect model. Therefore, there are two path hypotheses as follows, Path 1: Non-farming experience prompts new farmers to explore foreign markets, which indirectly affects their e-commerce participation behavior. Path 2: Non-farming experience prompts new farmers to operate brand building, and then choose e-commerce participation behavior. The existence of the above paths needs to be further discussed, and in this regard, the following is an empirical demonstration using the mediation effect model.

As depicted in Table 8, the non-farming experience of new farmers exerts a positive and statistically significant impact on both market orientation towards specific fields and agricultural product branding (Model 12 and Model 14). In Models 13 and 15, both variables related to the business status, namely the market-oriented type and agricultural product branding, significantly influence the e-commerce participation behaviors of new farmers at the 1% confidence level. Consequently, Hypothesis **H2** is corroborated. Furthermore, upon the inclusion of mediating variables, the non-agricultural work experience of new farmers continues to exert a positive and statistically significant influence on their e-commerce participation behaviors, as evidenced by the results of Model 13 and Model 15. Nevertheless, a notable decrease in the coefficients is observed. By referring to the approach put forward by Wen Zhonglin et al. [64] for verifying the significance of the mediating effect, it can be ascertained that both pathways associated with the agricultural business status exhibit partial mediating effects. Consequently, Hypothesis **H3** is effectively corroborated. First, new farmers facing the field market are more aware of the needs and preferences of the field market, and most of the agricultural products they produce and sell are further processed to add more added value to meet the needs of consumers in the field market, which makes them need to broaden their sales channels to realize economic benefits, and thus are more inclined to participate in the e-commerce sales of agricultural products. Brand is an important bridge connecting consumers and products, branded agricultural products means that the production process is more standardized, the quality of the product is guaranteed to improve product recognition, and enhance the consumer's willingness to buy and demand, and then need to expand the e-commerce sales channels to meet market demand. In summary, the mediating effect test validates Hypothesis H2 and H3. Non-farming experience not only directly impacts new farmers' e-commerce participation behavior but also indirectly exerts an influence through two business-status-related mediating variables.

**Table 8:** Mediating effect of non-farming experience on e-commerce participation behavior.

variable name	Model 11	Model 12	Model 13	Model 14	Model 15
	E-commerce participation behavior	Type of market	E-commerce participation behavior	Branding of agricultural products	E-commerce participation behavior
Non-Agricultural Practitioner Experience	1.603** (0.348)	1.961*** (0.488)	1.501* (0.331)	1.856*** (0.474)	1.452* (0.324)
Type of market			1.799*** (0.363)		
Branding of agricultural products					2.923*** (0.650)
control variable	Controlled	Controlled	Controlled	Controlled	Controlled
Pseudo R2	0.070	0.147	0.081	0.291	0.101

### 4.4. Robustness tests

This paper conducts robustness tests by replacing the model and replacing the sample size, and the results are shown in Table 9. First, the dependent variable in this paper is a dichotomous variable, and the binary probit model is re-estimated to estimate the equation (1) (Model 16); second, 85% of the valid questionnaires are randomly selected to form a new sample with a sample size of 486, and the effect of non-farming experience on the e-commerce participation behaviors of the new farmers is re-estimated (Model 17). As shown in Table 9, the results of the robustness tests all show that there is a significant positive effect of non-farming experience on the e-commerce participation behavior of new farmers, which is consistent with the previous section, indicating that the results of the previous study are robust.

**Table 9:** Robustness test regression results.

variable name	Model 16: probit regression		Model 17: logistic regression (85% sample)	
	ratio	standard error	Odds Ratio	standard error
Non-Agricultural Practitioner Experience	0.367***	0.131	1.777**	0.412
distinguishing between the sexes	0.190	0.128	1.278	0.282
(a person's) age	−0.022***	0.006	0.970***	0.011
political profile	0.081	0.121	1.224	0.260
Agricultural Insurance Awareness	−0.024	0.046	0.941	0.076
Participation in cooperatives	0.143	0.123	1.217	0.262
years of experience	0.007	0.008	1.017	0.015
Share of income from agriculture	0.433*	0.208	1.683	0.617
capital investment	0.159***	0.055	1.376***	0.135
labor input	−0.026	0.048	0.930	0.078
Availability of support	0.321**	0.145	1.400	0.363
Ease of access to policies	−0.104*	0.057	0.831*	0.083
constant term (math.)	−0.516	0.461	0.385	0.313
Area dummy variables	Controlled	–	Controlled	–
Pseudo R2	0.065		0.058	
Prob > chi2	0.000		0.000	

### 4.5. Heterogeneity analysis

To explore how regional and gender differences affect the results, this paper conducts a heterogeneity analysis. First, the research sites (Zhejiang, Jiangsu, Anhui, Henan, Sichuan, and Gansu) were divided into East, Middle, and West regions. As a result, the new farmers were divided into three regions of East, Central and West by region and explored whether the participation behavior of new farmers in agricultural e-commerce was heterogeneous due to regional differences. In addition, the new farmers were grouped by gender for regression, so as to analyze the role of the direct impact of non-farming experience on the e-commerce participation behavior of new farmers in the gender grouping. In Table 10, Model I shows the regression results of regional heterogeneity and Model II shows the regression results of gender heterogeneity.

#### 4.5.1. Regional heterogeneity.

There are significant differences in resource endowment, industrial structure and market demand in different regions [65], and these factors have different degrees of influence on new farmers' e-commerce participation behavior. The regression results of Model 1 in the table show that there is significant regional heterogeneity in the e-commerce participation behavior of new farmers, which is due to the following reasons: in the eastern coastal region, new farmers are mostly engaged in the cultivation and sale of fresh agricultural products such as fruits and vegetables, and need to broaden the sales channels to sell local specialty agricultural products. In addition, in comparison, the development of e-commerce in the eastern region is more mature so that the policy measures to support the development of rural e-commerce are also more complete, so they prefer e-commerce sales of agricultural products. In the central and western regions, new farmers are more inclined to sell bulk agricultural products, such as grain and livestock products, through the Internet, and local policies may help them find processors and dealers without the need for piecemeal e-commerce sales. Differences in business characteristics, local policies and traditional habits and values of new farmers in different regions have different degrees of impact on new farmers' e-commerce participation behavior.

#### 4.5.2. Gender heterogeneity.

In modern society, the rising social status of women is closely related to the popularization of the idea of gender equality, which makes the important role of women in decision-making in agricultural production increasingly prominent [66]. This makes the important role of women in decision-making in agricultural production more and more prominent. Moreover, there are differences in the sensitivity and acceptance of new technologies between men and women [67]. Based on the above discussion, it is of practical significance to study the impact of self-employment experience on the e-commerce participation behavior of two different groups of men and women from the perspective of gender differences. From the regression results of Model 17, the non-farming experience has a significant positive impact on the e-commerce participation behavior of male farmers. However, the performance of the female new farmer group shows heterogeneity, i.e., their e-commerce participation behavior is not significantly affected by non-farming experience. Therefore, it can be learned that male new farmers are more aware of the advantages of new agricultural e-commerce channels and are willing to learn more about them and participate in them. Female new farmers are more conservative, and their non-farming experience does not significantly increase their e-commerce participation behavior and willingness.

## 5. Conclusions and policy recommendations

### 5.1. Conclusions

Based on 572 field research data of new farmers in six provinces, Zhejiang, Jiangsu, Anhui, Henan, Sichuan, and Gansu, this study theoretically and empirically analyzes the impact of non-farming experience on e-commerce participation decision-making and degree as well as the influence mechanism of non-farming experience, business situation and new farmers' e-commerce participation behavior, and explores the robustness and heterogeneity of the results, with the following conclusions:

(1) New farmers' participation in agricultural e-commerce is not high, 55.60% of new farmers have participated or are participating in agricultural e-commerce, of which, more than 80% of new farmers' e-commerce participation is below 40%.
(2) Non-farming experience has a positive effect on new farmers' e-commerce participation behavior decision-making and participation degree. Compared with purely farming new farmers, the probability of the occurrence of e-commerce participation behavior of new farmers with non-farming experience increases by 13.5%, and their level of e-commerce participation increases by 5.5%.
(3) There are regional and gender differences in the influence of non-farming experience on the e-commerce participation behavior of new farmers. Male new farmers in the eastern region have a stronger influence on their e-commerce participation behavior due to their non-farming experience.
(4) The business status of new farmers also significantly affects the decision of e-commerce participation behavior, and there is a partial mediating effect of business status between non-farming experience and e-commerce participation behavior, i.e., non-farming experience optimizes the business status by means of building the brand of agricultural products and orienting to the foreign market, which then promotes e-commerce participation of new farmers.
(5) In terms of control variables: new farmers' e-commerce participation is also positively influenced by new farmers' individual characteristics, the policy environment, and factors in terms of capital inputs, such as the share of agricultural income and capital investment.

### 5.2. Policy recommendations

Based on the research data of 572 new farmers in six provinces, this study reveals the mechanism and regional heterogeneity of the influence of non-farming experience and business conditions on the e-commerce participation behavior of new farmers. The following policy recommendations closely rely on the empirical findings and use data to drive policy design and enhance policy precision and implementation effectiveness:

*First, activate the e-commerce empowerment effect of the non-farming experience in a targeted manner.*

The empirical results show that non-farming experiences can increase the probability of e-commerce participation of new farmers by 13.5% and the degree of participation by 5.5%. Based on this, it is necessary to build a precise talent attraction and experience transformation mechanism: on the one hand, for e-commerce industry talents, enterprise and public institution retirees, college student village officials, and other non-agricultural experience groups, formulate special subsidy policies, such as issuing business start-up funds, relying on local human resources and social services departments to establish a “talent pool of non-agricultural experience”, and push agricultural e-commerce business start-up cases in a targeted manner On the other hand, design “non-farming experience + agricultural e-commerce” integration training courses to transform urban retail management experience into online marketing skills for agricultural products, focusing on training for groups of new farmers under the age of 35 with more than three years of non-farming work experience. It focuses on the training of new farmers under the age of 35 with more than 3 years of non-farming work experience (the group's participation in e-commerce in the eastern region has increased significantly, p < 0.01), helping new farmers to obtain cutting-edge skills and technical knowledge in a timely manner, accurately solving the problems of production and operation, and enhancing the quality of new farmers while improving their operating efficiency, thus driving the development of regional agricultural e-commerce.

*Second, strengthen the intermediary conduction role of business conditions.*

Fully drive scientific and technological innovation and business model innovation, improve the efficiency and quality of agricultural production, and create a distinctive agricultural brand. Business conditions have a 32% mediating effect between non-farming experience and e-commerce participation, with brand building contributing 65% of the mediating path. Policies need to focus on brand standardization and international market development: set up and give one-time incentives for brands to new farmers who have obtained green food certification, and promote the construction of “one county, one product” regional public brand (e.g., Anhui Dangshan Pear, Sichuan Pujiang kiwifruit); set up an international market development fund, and give a rebate as a percentage of export value to agricultural e-commerce enterprises with a certain amount of annual export value, giving tax rebates as a percentage of the export amount, and relying on cross-border e-commerce platforms to establish “special zones for agricultural products going to sea”. Actively promoting the research and development, promotion and certification of agricultural technologies, guiding new farmers to make use of modern planting technologies and equipment, such as smart agricultural systems and unmanned aircraft plant protection, to improve crop yields and quality, and promoting the standardization of agricultural production and operation, so as to achieve high-quality development of agricultural products and industries.

*Third, differentiated responses to regional and gender heterogeneity.*

The off-farm experience effect of male new farmers in the eastern region is 48% higher than that of women in the western region. When formulating policies, local governments need to fully consider the natural and social environments of different regions, as well as the differences between different individual new farmers. Maintaining farmers' interests, promoting common prosperity, and guiding a new generation of high-quality farmers to participate in the “Digital Commerce for Agriculture” project. Adopt corresponding incentives and guidance policies for new farmers under different resource endowment conditions, and create agricultural e-commerce products with regional characteristics according to market demand, regional classification guidance needs to be implemented: in the eastern region, focusing on male new farmers to build an “e-commerce industrial park + cold chain warehousing” all-in-one park, and piloting the In the eastern region, focus on male new farmers to build “e-commerce industrial park + cold chain storage” integrated park, pilot “e-commerce business loan” credit loan model; in the central and western regions, targeting female new farmers to carry out the “Digital Woman” training program, the development of mobile e-commerce operation guide, the promotion of the “cooperatives+female leaders” model.

*Fourth, optimize the monitoring and reach of policy implementation effects.*

Establish a dynamic monitoring system between policy effects, e-commerce participation, and new farmers' income increase, tracking the conversion rate of non-farming experience talents, brand-building effectiveness, and policy yield, and optimizing policy tools with data feedback to realize the rural revitalization practice of “data-driven decision-making and precise targeting of policies”. In addition, the policy and infrastructure protection function is consolidated. For every 1-unit increase in the policy awareness of new farmers, e-commerce participation increases by 4.2%; for every 10% decrease in the share of agricultural income, participation decreases by 2.8%. Therefore, it is necessary to optimize the policy reach and capital investment mechanism: disseminate the policy through the dual line of ‘rural radio + short video', produce the ‘Agricultural E-commerce Policy Illustrated Handbook', and set up a policy consultant at the village level; provide subsidies for the purchase of e-commerce equipment for new farmers with an agricultural income of less than 30%, and guide non-farming incomes to feed agricultural e-commerce.

### 5.3. Practical significance and limitations

#### 5.3.1. Practical significance of the research.

In terms of agricultural policies, the findings of this study suggest that the government should intensify its support for new farmers. For instance, providing more e-commerce training programs tailored to new farmers to enhance their e-commerce operation capabilities echoes the policy implications of strengthening the attraction of talent to return to rural areas and creating a favorable policy environment for rural e-commerce of agricultural products. Meanwhile, when formulating policies, regional and individual heterogeneities should be fully considered. Differentiated policies should be formulated according to the characteristics of different regions. For example, in eastern regions, e-commerce support policies should be further refined, while in central and western regions, emphasis should be placed on strengthening the construction of logistics infrastructure to promote the balanced development of agricultural product e-commerce.

Regarding rural development, encouraging new farmers to explore external markets and promoting the branding of agricultural products can drive rural economic development, increase farmers' income, and facilitate the implementation of the rural revitalization strategy. For example, the policy implication of promoting the high-quality development of agricultural products and leading the branding of agricultural products can enhance the market competitiveness of agricultural products, expand sales channels, and achieve high-quality development of agriculture.

#### 5.3.2. Research limitations.

Despite the fact that this study used a variety of methods to ensure the reliability of the findings, there are still some limitations. In terms of sample selection, only new farmers in six provinces, Zhejiang, Jiangsu, Anhui, Henan, Sichuan, and Gansu, were selected as samples for this study. This may not fully represent the situation of new farmers in the whole country, resulting in sample selection bias. Future research could expand the sample to cover new farmers in more regions.

In terms of the variable selection, although numerous influencing factors were considered, some potentially important variables may have been overlooked. For example, the influence of new farmers' family backgrounds and social networks on their e-commerce participation behavior was not considered. Subsequent studies can further improve the variable system and provide a more comprehensive analysis of the influencing mechanisms. In the limitation of methodology, the study constructed Logistic model and Tobit model based on cross-sectional data, which could not clarify the time-sequential relationship between variables.The Tobit model analysed the degree of participation, which could not capture the dynamic mediation path of ‘non-farming experience→business situation→e-commerce participation'. Future research can adopt panel data, and panel data using a fixed-effects model can better track how annual changes mediate the non-farm experience effect over time, and explore in depth the changing patterns of non-farm work experience, business level, and e-commerce participation behaviors of new farmers over time.
